# Association Between Mechanical, Physiological, and Technical Parameters With Canoe Slalom Performance: A Systematic Review

**DOI:** 10.3389/fphys.2021.734806

**Published:** 2021-11-18

**Authors:** Leonardo Henrique Dalcheco Messias, Ivan Gustavo Masselli Dos Reis, Viktor Bielik, Ana Luíza Paula Garbuio, Claudio Alexandre Gobatto, Fúlvia Barros Manchado-Gobatto

**Affiliations:** ^1^Laboratory of Multidisciplinary Research, São Francisco University, Bragança Paulista, Brazil; ^2^Department of Biological and Medical Sciences, Faculty of Physical Education and Sports, Comenius University, Bratislava, Slovakia; ^3^Laboratory of Applied Sport Physiology, School of Applied Sciences, University of Campinas, Limeira, Brazil

**Keywords:** water sports (MeSH), power, force, metabolism, physiology, biomechanics

## Abstract

This study aimed to systematically review studies that evaluated and compared mechanical, physiological, and technical parameters with the performance of slalom athletes. PubMed, SPORTDiscuss, and Scopus databases were searched until September 10, 2021, with no restriction of published data. The Preferred Reporting Items for Systematic Reviews and Meta-Analyses guided the study's screening and quality assessment performed by an external reviewer using a 16-checklist item. A search of the databases identified 125 studies, but only eight were eligible, including a total sample of 117 male athletes. Four reports only associated mechanical or technical parameters with the performance of the paddler. Concerning the remaining studies, only one correlated physiological data, and the others associated more than one parameter with race time. Most of the eligible reports presented significant associations between mechanical/physiological components and slalom performance. Eligible studies support that high-force development during a slalom race is a relevant parameter for performance. Aerobic metabolism is highly required during slalom tasks and is inversely associated with race time, although it may not increase the chances of winning medals. Few reports have associated canoe slalom performance with technical components, and further research should focus on this matter.

## Introduction

In North American history, canoeing played a critical role for commercial purposes; however, it was overshadowed by the construction of transcontinental railways (Shephard, 1987). Nonetheless, interest in canoeing as a sport continues to increase, and the International Canoe Federation (ICF) currently considers more than 10 canoeing disciplines, with the sprint and slalom challenges in the Summer Olympic Games (ICF, 2021). While sprint takes place on flatwater courses, the slalom discipline occurs in natural or artificial rivers, also called whitewater. This “simple” difference does not only drastically affect the specificity of the sport, but the number of scientific reports in each. While considerable research has been conducted on sprint athletes (Shephard, 1987; Michael et al., 2008, 2009), there are few studies on canoe slalom athletes.

Slalom courses are inconsistent in terms of obstacles, routes, and gates, offering large performance variability (Nibali et al., 2011). Canoe slalom athletes must negotiate courses with a maximum of 25 gates, including upstream and downstream gates, and an approximate length of 300 m. The challenge includes a competitive course with eddies, waves, and stoppers. Therefore, it is clear that canoe slalom complexity is purposeful rather than chance. Such factors directly impact scientific studies designed to understand the relevant factors underlying canoe slalom athlete performance. Thus, the science of this sport is challenging. Moreover, the Olympic Games comprise K1 (kayak single) and C1 (canoe single) classes. In addition, the canoe double category (C2) is challenged at the international level. In canoe (both C1 and C2), a single-blade paddle is used by the athlete while their legs are maintained at the knees and tucked under their body. In the kayak category, the double-bladed paddle is used, and the athlete is kept in a seated position in the boat (ICF, 2021).

Studies published until 2010 have triggered relevant discussions on energy metabolism during races (Sidney and Shephard, 1973; Shephard, 1987; Zamparo et al., 2006), as well as the biomechanical (Hunter et al., 2007, 2008; Hunter, 2009), and psychological aspects of slalom athletes (Males et al., 1998; Moran and MacIntyre, 1998; White and Hardy, 1998; MacIntyre et al., 2002; Macintyre and Moran, 2007). Zamparo et al. (2006) verified that both aerobic and anaerobic metabolism are relevant during slalom tasks. Moreover, strokes performed during competitions were properly addressed (Hunter et al., 2007), and strategies to negotiate upstream gates have been discussed in detail (Hunter et al., 2008). However, among these studies, few have compared and/or associated the collected results with slalom performance (Hunter et al., 2008).

In a later narrative review, we initiated a discussion on this matter (Messias et al., 2014), but the lack of published studies until that moment precluded deeper inferences. Since then, research groups have tried to identify the relevant components associated with canoe slalom athlete performance. Slalom tasks require great physical fitness and precise technical skills (Messias et al., 2014). Therefore, mechanical, physiological, and technical components play important roles in canoe slalom races. However, no systematic review has focused on demonstrating which of these parameters are associated with canoe slalom performance. In this way, the present manuscript aimed to systematically review studies that evaluate and compare mechanical, physiological, and technical parameters with the performance of slalom athletes.

## Materials and Methods

### Search Strategy

PubMed, SPORTDiscuss, and Scopus databases were searched until September 10, 2021, with no restriction of published data. “AND,” “OR” and “NOT” operators were applied to terms such as “canoe slalom OR slalom kayaking OR slalom canoeing” AND “aerobic OR anaerobic OR mechanical OR power OR force OR strength OR velocity OR neuromuscular OR physiological OR technical OR performance.” Reference lists and citations from studies involving canoe slalom were manually searched.

### Eligibility Criteria and Meticulous Inclusion/Exclusion Criteria

The Preferred Reporting Items for Systematic Reviews and Meta-Analyses (PRISMA) (Moher et al., 2009) was adopted to guide the screening of studies associating or comparing the performance of slalom athletes. Inclusion criteria were: (a) studies published in English; (b) cross-sectional reports associating a canoe slalom athlete's performance with mechanical, physiological, or technical parameters; (c) studies evaluating a canoe slalom athlete's performance in tasks where technical implements were designed; (d) reports comparing canoe slalom athletes' results from national and international competitions according to mechanical, physiological, or technical variables; (e) studies where canoe slalom athletes' performances were measured under specific conditions (i.e., on flatwater or whitewater); and (f) where the methodologies of the mechanical, physiological, or technical assessments were properly presented and explained. Published studies were not included if: (a) only the abstract was provided; (b) there was a lack of information on the statistical procedures adopted for associating/comparing the results; (c) studies including athletes from other canoeing modalities that did not present a separate group/analysis for only canoe slalom athletes; and (d) reports that only characterized mechanical, physiological, or technical rather than associating these variables with performance.

### Data Extraction

Two reviewers (LM and AG) independently screened the manuscripts. Title and abstracts were screened first, and then only full texts of studies that passed this stage were checked. Disagreements between these were sent to a third reviewer (IR) and these were resolved by consensus. The extracted data consisted of: (a) sample characteristics, including anthropometrics, body composition, and age; (b) slalom category (K1, C1, C2); (c) protocols for mechanical, physiological, and technical assessments; (d) details of the slalom performance task; and (e) statistical results from comparisons between slalom athletes or associations between performance and mechanical, physiological, or technical variables. Further results that were not related to the aim of this systematic review were not presented or discussed.

### Quality Assessment

Tools for quality assessment of studies included in systematic reviews are available for distinct scientific reports (Downs and Black, 1998; CRD, 2009; Wells et al., 2021). However, these may not be suitable for the included studies in this review, which are mostly cross-sectional and analytical studies on the associations between performance and mechanical, physiological, or technical variables. Therefore, we opted for a 16-item checklist previously conducted in systematic reviews concerning soccer (Sarmento et al., 2018a,b; Low et al., 2020). This checklist includes the study purpose (1), proper literature background (2), appropriate design (3), sample details (4), sample size justification (5), informed consent (6), reliability of the measured outcomes (7), validity of the measured outcomes (8), method details (9), presented results in terms of statistical significance (10), appropriate analysis methods (11), reported practical importance (12), report of drop-outs (13), appropriate conclusions (14), practical applications (15), and limitations of the study (16). Each question was scored on a binary scale of 0 (no) and 1 (yes), except for questions 6 and 13 that also include “If not applicable, assume 3.” All answers were summed, and the final score was divided by the maximum that a study could reach (i.e., 16) and expressed as a percentage. The quality classification was conducted as follows: (a) low methodological quality refers to a score ≤ 50%; (b) good methodological quality lies between 51 and 75%; and (c) excellent methodological quality refers to >75% (Sarmento et al., 2018a). Considering that the proponents of this systematic review are also authors of some of the eligible studies, the quality assessment was performed by an external reviewer with experience in the sports science field.

## Results

### Search and Quality Assessment

**Figure 1:**
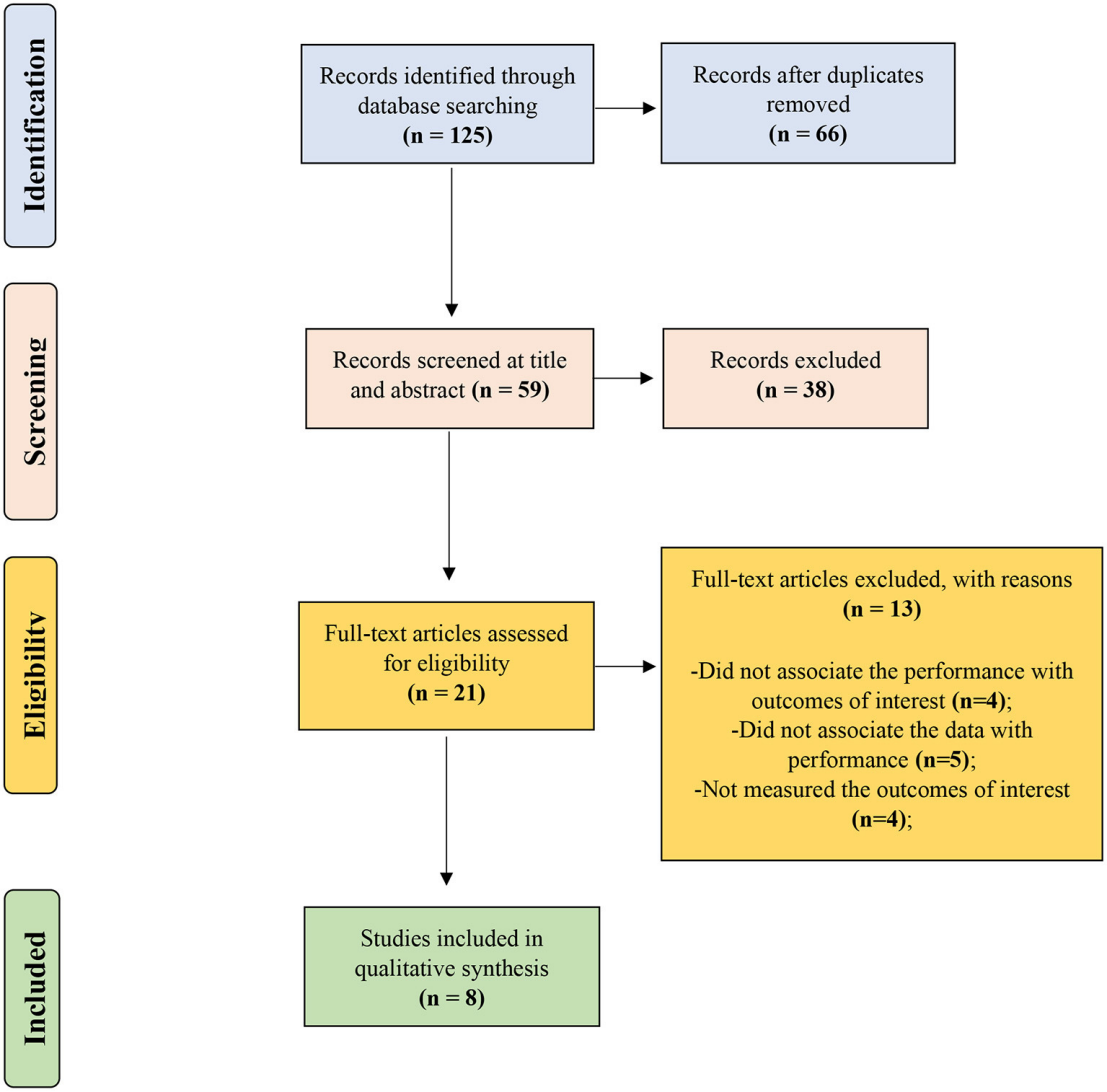
Flow diagram of the Preferred Reporting Items for Systematic Reviews and Meta-Analyses (PRISMA).

A search of the databases identified 125 studies. After the removal of duplicates (66 studies), 59 articles were checked at the title and abstract level, of which 38 were excluded. Finally, 21 full-texts were screened, and eight (Hunter et al., 2008; Messias et al., 2015; Vieira et al., 2015; Ferrari et al., 2017; Bielik et al., 2019, 2020; Macdermid et al., 2019; Baláš et al., 2020) reached the eligibility criteria (Figure 1). The eligible study's mean quality was high (92.9 ± 2.9%) and classified as having excellent methodological quality according to the 16-item checklist.

### Athlete Characteristics and Slalom Modalities

Overall, 117 male slalom athletes were evaluated, and their performance was associated with mechanical, physiological, or technical variables (Table 1). The canoe slalom athletes were from Europe and South America, with Slovak comprising 61 athletes, followed by Brazil with 30 and Czech Republic and New Zealand with 18 and 8 athletes, respectively. Only one study evaluated Olympic medalists (Bielik et al., 2019), while others tested elite or competitive canoe slalom athletes. One study evaluated athletes from the C1 category (Baláš et al., 2020), while the other four studies included the K1 category (Messias et al., 2015; Vieira et al., 2015; Ferrari et al., 2017; Macdermid et al., 2019). The remaining three tested athletes from K1/C1 (Hunter et al., 2008) and K1, C1, and C2 (Bielik et al., 2019, 2020). Body mass and height were measured in 87.5% of the included studies (72.6 ± 4.8 kg; 177.6 ± 3.5 cm), and body fat was calculated in three reports (10.2 ± 1.4 %). Age was presented in 75% of the eligible studies (21 ± 4 years). Hunter et al. (2008) focused on the 10 fastest runs from men's kayak, woman's kayak, and men's canoe rather than on athletes' characteristics.

**Table 1:** Characteristics of the eligible studies.

**Study**	**Sample**	**Age, body composition, or anthropometrics**	**Canoe slalom modality**	**Outcome of interest**
Hunter et al. (2008)	^*^	^#^	K1, C1	Technical
Messias et al. (2015)	*N* = 12 males—Elite (Brazilian national team)	Age = 18 ± 2 years Body mass = 68.1 ± 0.6 kg	K1	Mechanical
		Height = 174 ± 1 cm		
		Body fat = 10.3 ± 0.1 %		
Vieira et al. (2015)	*N* = 6 males—High-performance (5 classified the top eight in the Brazilian Canoe Confederation national rankings)	Age = 17 ± 2 years Body mass = 68.0 ± 5.0 kg Height = 175 ± 6 cm	K1	Technical, physiological
Ferrari et al. (2017)	*N* = 12 males—Elite (Brazilian national team)	Age = 18 ± 2 years Body mass = 68.1 ± 0.6 kg Height = 174 ± 1 cm	K1	Physiological
Bielik et al. (2019)	*N* = 42 males—Olympic medalists (*N* = 6) and non-Olympics (*N* = 36) from Slovak national team	*Olympic Medalists* Age = 22 ± 1 years Body mass = 76.6 ± 1.3 kg	K1, C1, C2	Physiological, mechanical
		Height = 181 ± 1 cm		
		Body fat = 9.6 ± 0.7 %		
		*Non-Olympics*		
		Age = 32 ± 3 years		
		Body mass = 82.2 ± 5.9 kg		
		Height = 184 ± 9 cm		
		Body fat = 9.4 ± 2.0 %		
Macdermid et al. (2019)	*N* = 8 males—Competitive (of the New Zealand Slalom development team)	Body mass = 65.8 ± 6.0 kg Height = 173 ± 4 cm	K1	Mechanical
Baláš et al. (2020)	*N* = 18 males—High performance elite athletes of international level (*N* = 9) and elite athletes of national level (*N* = 9) from Czech Republic	*High performance* Age = 24 ± 7 years Body mass = 75.2 ± 5.6 kg	C1	Technical
		Height = 180 ± 4 cm		
		*Elite*		
		Age = 19 ± 4 years		
		Body mass = 72.5 ± 4.4 kg		
		Height = 180 ± 2 cm		
Bielik et al. (2020)	*N* = 19 males—Medalists (*N* = 11) and non-medalists (*N* = 8) at the Junior/U23 European and World Championships from Slovak national team	*Medalists* Age = 18 ± 1 years Body mass = 75.4 ± 4.6 kg	K1, C1, C2	Physiological, mechanical
		Height = 176 ± 4 cm		
		Body fat = 12.9 ± 2.3 %		
		*Non-medalists*		
		Age = 19 ± 1 years		
		Body mass = 73.6 ± 6.3 kg		
		Height = 179 ± 4 cm		
		Body fat = 8.9 ± 3.0 %		

* *Authors used the number of races as samples rather than athletes*.

# *Data not presented in the study*.

*K1, kayak single; C1, canoe single; C2, canoe double*.

### Performance Measurements

Three studies used simulated races in whitewater (Messias et al., 2015; Vieira et al., 2015; Ferrari et al., 2017) and two in flatwater (Macdermid et al., 2019; Baláš et al., 2020) courses. Two studies (Messias et al., 2015; Ferrari et al., 2017) inserted 24 gates (18 downstream and 6 upstream) during the performance task in whitewater, while Vieira et al. (2015) adopted only 11 (eight downstream and three upstream). In flatwater trials, the slalom athletes from the study by Macdermid et al. (2019) traversed 15 gates, with 11 downstream and 4 upstream gates. The course proposed by Baláš et al. (2020) had buoys for pivot turns rather than gates. Finally, the remaining studies adopted, as performance indices, the results from the Beijing 2008, London 2012, or Rio 2016 Summer Olympic Games (Bielik et al., 2019), the 2019 Junior/U23 European and World championships (Bielik et al., 2020), and the 2005 World Championship (Hunter et al., 2008).

### Mechanical Variables and Slalom Performance

Two studies only associated mechanical parameters with performance (Messias et al., 2015; Macdermid et al., 2019). The force results (peak, mean, minimum, and impulse) from Messias et al. (2015) were measured by a 30-s tethered all-out effort in a swimming pool, and inverse but significant relationships between these data and the simulated race time were observed. Macdermid et al. (2019) also measured the force parameters during a simulated task. However, although a moderate to strong correlation between race time and peak force/rate of peak force development was observed, these were not significant. Bielik et al. (2019) compared the power (paddling) and velocity (running) at maximum oxygen uptake (VO_2max_) from Slovakian medalists and non-medalists in Olympic games and observed considerable raw differences in Rio 2016 for paddling and London 2012 for running. The same group observed similar mean power on bench press/pull and velocity at VO_2max_ between medalists and non-medalists in the 2018/2019 Junior/U23 European and World championships (Bielik et al., 2020) (Table 2).

**Table 2:** Methodological aspects and main results from studies that associated the slalom performance with mechanical parameters or compared mechanical data between athletes with distinct canoe slalom performances.

**Study**	**Testing procedure**	**Mechanical measurements**	**Specifications of the performance task**	**Overview of the main outcomes**
Messias et al. (2015)	All-out 30-sec test in tethered canoe system	Absolute and relative peak, mean, and minimum forces, besides impulse	Simulated race in a white-water course with 24 gates (18 downstream 6 upstream)	(a) Race time and absolute peak force: *r* = −0.60; *p* = 0.038; (b) Race time and relative peak force: *r* = −0.71; *p* = 0.008; (c) Race time and absolute mean force: *r* = −0.61; *p* = 0.033; (d) Race time and relative mean force: *r* = −0.73; *p* = 0.006; (e) Race time and absolute impulse: *r*= −0.61; *p* = 0.034; (f) Race time and absolute impulse: *r* = −0.73; *p* = 0.005.
Bielik et al. (2019)	Incremental running and paddling test	Power and velocity at VO_2max_	Results from Beijing 2008, London 2012, and Rio 2016 Olympics	(a) Raw difference of 9 W between power at VO_2max_ of non-medalists (146 ± 42 W) and Slovakian medalists (155 W) at Beijing 2008; (b) Raw difference of 26 W between power at VO_2max_ of non-medalists (136 ± 32 W) and Slovakian medalists (110 ± 14 W) at Rio 2016; (c) Raw difference of 1.4 km·h^−1^ between velocity at VO_2max_ of non-medalists (19.4 ± 1.3 km·h^−1^) and Slovakian medalists (18.0 ± 0.0 km·h^−1^) at London 2012; (d) Raw difference of 0.2 km·h^−1^ between velocity at VO_2max_ of non-medalists (19.1 ± 0.4 km·h^−1^) and Slovakian medalists (19.3 ± 3.5 km·h^−1^) at Rio 2016.
Macdermid et al. (2019)	Simulated race using the kayak power meter	Stroke length, impulse, peak force, time to peak force	Simulated race in flat-water comprising 15 gates (11 downstream 4 upstream)	(a) Race time and peak force slope *R*^2^ = 0.40; *p* = 0.091; (b) Race time and peak force y-intercept *R*^2^ = 0.35; p = 0.117; (c) Race time and rate of peak force development slope: *R*^2^ = 0.41; *p* = 0.084; (d) Race time and rate of peak force development y-intercept: *R*^2^ = 0.36; *p* = 0.115.
Bielik et al. (2020)	Maximal bench press and bench pull testing and incremental running test	Absolute and relative mean power of the concentric phase and velocity at VO_2max_	Results from 2018 and 2019 Junior/U23 European and World championships	(a) Absolute mean power on bench press: *p* = 0.688; (b) Relative mean power on bench press: *p* = 0.454; (c) Absolute mean power on bench pull: *p* = 0.847; (d) Relative mean power on bench pull: *p* = 0.656; (e) Velocity at VO_2max_: *p* =0.975.

*VO_2max_, maximum oxygen uptake*.

*he raw differences between groups in the study of Bielik et al. (2019) are not indicative of statistical difference*.

*The p values of Bielik et al. (2020) refers to post-hoc analysis from the two-way ANOVA (canoe slalom medalists vs canoe slalom non-medalists)*.

### Physiological Variables and Slalom Performance

Vieira et al. (2015) observed a moderate to strong inverse correlation between physiological parameters measured during (i.e., maximum, mean, and minimum heart rate) or after (i.e., blood lactate concentration peak) the simulated task and the race time, although this was not significant. Ferrari et al. (2017) measured the critical force and maximal lactate steady state intensity (MLSS) in a tethered ergometer and observed an inverse and significant relationship only between race time and MLSS. In the study by Bielik et al. (2019), the aerobic power of Slovakian medalists at the Rio 2016 and London 2012 Olympic games was consistently lower (i.e., raw data) than that of the rest of the team (i.e., non-medalists). Moreover, similar VO_2max_ was observed between Slovakian Junior/U23 medalists and non-medalists in the 2018 and 2019 European and World championships (Table 3).

**Table 3:** Methodological aspects and main results from studies that associated the slalom performance with physiological parameters or compared physiological data between athletes with distinct canoe slalom performances.

**Study**	**Testing procedure**	**Physiological measurements**	**Specifications of the performance task**	**Overview of the main outcomes**
Vieira et al. (2015)	Physiological measurements throughout and after two simulated races	HR during races and [Lac] after races	Two white-water simulated races comprising 11 gates (8 downstream and 3 upstream)	First simulated race^#^ (a) Race time and [Lac] peak: *r* = −0.46; *p* = 0.349; (b) Race time and maximum HR: *r* = −0.66; *p* = 0.338; (c) Race time and mean HR: *r* = −0.69; *p* = 0.301; (d) Race time and minimum HR: *r* = −0.37; *p* = 0.625; Second simulated race^#^ (a) Race time and [Lac] peak: *r* = −0.02; *p* = 0.967; (b) Race time and maximum HR: *r* = −0.53; *p* = 0.269; (c) Race time and mean HR: *r* = −0.73; *p* = 0.096; (d) Race time and minimum HR: *r* = −0.62; *p* = 0.183.
Ferrari et al. (2017)	CF test and MLSS protocol on tethered ergometer	CF from linear and hyperbolic models and MLSS intensity	Simulated race in a white-water course with 24 gates (18 downstream 6 upstream)	(a) Race time and MLSS intensity: *r* = −0.67; *p* = 0.016; (b) Race time and CF linear: *r* = −0.41; *p* = 0.180; (c) Race time and CF hyperbolic: *r* = −0.48; *p* = 0.106.
Bielik et al. (2019)	Incremental running and paddling test	VO_2max_	Results from Beijing 2008, London 2012, and Rio 2016 Olympics	(a) Raw difference of 0.7 ml·kg^−1^·min^−1^ between the VO_2max_ on paddling of non-medalists (47.1 ± 7.3 ml·kg^−1^·min^−1^) and Slovakian medalists (47.8 ml·kg^−1^·min^−1^) at Beijing 2008; (b) Raw difference of 4.4 ml·kg^−1^·min^−1^ between the VO_2max_ on paddling of non-medalists (47.6 ± 6.6 ml·kg^−1^·min^−1^) and Slovakian medalists (43.2 ± 3.1 ml·kg^−1^·min^−1^) at Rio 2016; (c) Raw difference of 8.3 ml·kg^−1^·min^−1^ between VO_2max_ on running of non-medalists (60.6 ± 7.1 ml·kg^−1^·min^−1^) and Slovakian medalists (52.3 ± 1.7 ml·kg^−1^·min^−1^) at London 2012; (d) Raw difference of 8.1 ml·kg^−1^·min^−1^ between VO_2max_ on running of non-medalists (60.4 ± 6.2 ml·kg^−1^·min^−1^) and Slovakian medalists (52.3 ± 6.8 ml·kg^−1^·min^−1^) at Rio 2016.
Bielik et al. (2020)	Incremental running test	VO_2max_	Results from 2018 and 2019 Junior/U23 European and World championships	Similar VO_2max_ between medalists and non-medalists (*p* = 0.609)

# *Results not presented in the published study but informed by authors*.

*HR, heart rate; [Lac], blood lactate concentration; CF, critical force; MLSS, maximal lactate steady state; VO_2max_, maximum oxygen uptake*.

### Technical Variables and Slalom Performance

Three studies associated technical data from slalom athletes and their performance (Table 4). Hunter et al. (2008) verified weak, moderate, and strong correlations between race time and stroke time, stroke length, and time spent with the blade in the water, respectively. However, the authors did not demonstrate the significance of these relationships. Vieira et al. (2015) provided a significant correlation between the number of paddles against the current and race time in the first simulated race. In the second performance trial, these authors observed significant correlations between the total number of paddles, paddles with the current, completed cycles of paddling, and mean velocity with the athlete's performance. Baláš et al. (2020) showed that high-performance elite slalom athletes at international levels performed better in trials over 40, 80, and 200 meters compared to those at the national level. Notably, the 200 m had the greatest difference between the levels of athletes.

**Table 4:** Methodological aspects and main results from studies associating the slalom performance with technical parameters.

**Study**	**Testing procedure**	**Technical measurements**	**Specifications of the performance task**	**Overview of the main outcomes**
Hunter et al. (2008)	Footage of semi-finals and finals runs of the 2005 World Championship	Stroke time, stroke count, and time spent with the blade in the water^*^	Results from the 2005 World Championship	(a) Race time and stroke time: *r* = −0.14; (b) Race time and stroke count: *r* = 0.45; (c) Race time and time spent with the blade in the water: *r* = 0.72.
Vieira et al. (2015)	Technical measurements throughout two simulated races	Total number of paddles (Total._Paddles_), paddling with the current (Pad._With_), paddling against current (Pad._Against_), complete cycle of paddling (Compl._Cycle_), cross paddling (Cross._Paddling_), and mean velocity	Two whitewater simulated races comprising 11 gates (8 downstream and 3 upstream)	First simulated race^#^ (a) Race time and Total._Paddles_: *r* = 0.55; *p* = 0.257; (b) Race time and Pad._With_: *r* = 0.04; *p* = 0.929; (c) Race time and Pad._Against_: *r* = 0.87; *p* = 0.022; (d) Race time and Compl._Cycle_: *r* = 0.43; *p* = 0.387; (e) Race time and Cross._Paddling_: *r* = 0.36; *p* = 0.477; (f) Race time and mean velocity: *r* = −0.68; *p* = 0.132. Second simulated race^#^ (a) Race time and Total._Paddles_: *r* = 0.86; *p* = 0.025; (b) Race time and Pad._With_: *r* = 0.84; *p* = 0.033; (c) Race time and Pad._Against_: *r* = 0.70; *p* = 0.118; (d) Race time and Compl._Cycle_: *r* = 0.91; *p* = 0.010; (e) Race time and Cross._Paddling_: *r* = 0.28; *p* = 0.589; (f) Race time and mean velocity: *r* = −0.84; *p* = 0.036.
Baláš et al. (2020)	Time paddling tests (3 × 40 m, 80 m, 200 m) with a different number of pivot turns	–^§^	Performance trials over 40, 80, and 200 meter in flat water with visible buoys for performing pivot turns, except for one 40 m trial	(a) 40 m without pivoting: *p* = 0.047 (b) 40 m with pivoting: *p* = 0.001 (c) 80 m with pivoting: *p* = 0.005 (d) 200 m with pivoting: *p* < 0.001

* * Note that this study measured other technical aspects, but only these were correlated with the performance*.

# *Results not presented in the published study but informed by authors*.

§ *The pivot technical elements were added into each trial rather than evaluated*.

## Discussion

This systematic review showed that mechanical, physiological, and technical components may affect the performance of canoe slalom athletes. Nonetheless, despite the increase in the scientific interest of this sport over the last 7 years, more studies are necessary to understand the demands of slalom trials and improve the assessment of these parameters with increased ecological validity. Regarding the performance measurement, great variability in terms of course, gates, and obstacles were identified among the eligible studies. This is not surprising, since slalom championships are projected to be inconsistent, offering a great challenge for athletes, coaches, and researchers.

With respect to mechanical parameters, Messias et al. (2015) showed inverse correlations between race time and force (peak, mean, and impulse) from a maximal 30-s all-out test. This ergometer was proposed to preserve forward stroke characteristics while measuring the technical and/or physiological parameters. Additionally, the tethered system was suggested as an alternative training tool that is not affected by the climatic conditions, since it can be used in a swimming pool. Macdermid et al. (2019) assessed slalom athletes in a flatwater course with a kayak paddle shaft (Macdermid and Fink, 2017) and suggested that peak force and its rate of development are relevant during races, although correlations between these parameters and race time were not significant. Moreover, these authors visualized that impulse and the time to develop peak force per stroke remained similar throughout the performance task, while the peak force magnitude decreased. Hence, both Messias et al. (2015) and Macdermid et al. (2019) corroborate that a high stroke force level is important for slalom race performance.

Studies not eligible for this systematic review may shed some light on these aspects. The early work of Sidney and Shephard (1973) suggested that the trunk muscles and upper extremities are engaged in rhythmic work during slalom, which may depend more on cardiorespiratory power rather than strength. We believe that over the last 40 years, the canoe slalom has evolved and strength training has gained higher importance, as has been found in other sports (Hebert-Losier et al., 2014, 2017; Styles et al., 2016; Kniffin et al., 2017). However, the mean power of bench pulls and presses appears to have a deeper influence on canoe sprint as opposed to slalom (Bielik et al., 2020). Canoe slalom paddlers use narrower hand grips, which results in shorter, more powerful strokes with increased elbow flexion than that reported for a top-level sprint (Zahalka et al., 2011). The narrower hand-grip of these athletes strongly engages the arm muscles to produce rapid movement. Conversely, hand-grip of canoe sprint athletes primary engage the trunk muscles (McKean and Burkett, 2014).

Research on the physiological parameters of slalom athletes has provided important data for coaches and athletes. Except for the incremental running test in two studies (Bielik et al., 2019, 2020), all reports adopted distinct ergometers and tools to assess the physiological parameters of slalom athletes. The weak and non-significant correlations of Vieira et al. (2015) regarding heart rate/blood lactate and race time can be explained by the low number of athletes tested in this study. Ferrari et al. (2017) have confirmed the importance of aerobic metabolism in slalom athletes, suggesting that the oxidative system may indirectly affect performance by improving the training load and leading to faster recovery between races. In summary, slalom races require great anaerobic participation during paddling efforts in order to surpass obstacles (Baker, 1982; Messias et al., 2015; Vieira et al., 2015). On the other hand, the aerobic metabolism demand is also considerable (Zamparo et al., 2006) and related to performance (Ferrari et al., 2017). These factors, however, may not increase the chances of winning medals according to Bielik et al. (2019, 2020).

Although the technical aspects of strokes performed by slalom athletes have been described in detail (Hunter et al., 2007), few studies have assessed the relevance of these for performance. Hunter et al. (2008) verified in the top 10 runs of the 2005 World Championship that 67–71% of the strokes performed were in the forward direction, while 30% of strokes were performed to turn the boat. These authors also noticed that race time and the total time that athletes spent with their blades in the water were positively correlated, which was expected. However, the same was not observed for the percentage of time the paddlers spent with the blade in the water, suggesting that regardless of race time, this percentage was unchanged. Vieira et al. (2015) tracked the strokes performed during two simulated races, and their results suggest that athletes may vary their strategy from race to race even in an identical course. Such results strengthen the idea of a purposeful challenge offered by this sport to coaches and athletes. Therefore, further studies on the paddler's strategy and performance are necessary.

Baláš et al. (2020) proposed an alternative way to verify the influence of a specific skill on the athlete's performance over a flatwater course. Pivot turns were added in the time paddling tests over 40, 80, and 200 m. Interestingly, high-performance athletes at the international level presented better results in every trial than those at the national level. This result adds to the previous discussion on the measurements to be considered by coaches when evaluating performance enhancement. The course proposed by these authors can be easily reproduced, and further studies are also required to create a group of trials that can offer insights on performance by measuring other technical skills. In addition, identifying associations with these results in simulated trials or situations mirroring real slalom conditions can strengthen the importance and use of such assessments. These should consider the rules provided by the ICF, such as a maximum of 25 gates and a length of 300 m. In this scenario, canoe slalom athletes should accomplish the race between 90–110 s and require great force, velocity, and power development in addition to anaerobic metabolism.

The athletes evaluated in the eligible studies were mostly men. Only the investigation by Hunter et al. (2008) also considered slalom runs in female athletes. Further research with females' slalom athletes is encouraged, especially because female K1 and C1 classes are challenged in the Olympic Games. Another interesting fact noted in this systematic review was that slalom athletes from only four countries were tested. It must be recognized that Slovakia, the Czech Republic, and New Zealand won medals at the last Summer Olympic Games. Still, other countries from Europe and Oceania have also won medals in both Olympic and World Championship tournaments. Accordingly, to improve the scientific knowledge of slalom athletes, further studies are required with athletes from different nationalities. In addition, none of the eligible studies provided a deep discussion on the performance of the different classes (i.e., kayak and canoe). Hunter et al. (2007) concluded that athletes in the C1 category may perform fewer strokes during races than K1 athletes, but this does not impact performance time. The studies included in this systematic review did not compare these parameters between classes, precluding a thorough discussion on this matter. However, further research comparing the physiological, biomechanical, and technical aspects of classes is strongly encouraged.

A further point worth discussing is the variability in performance measurements. Although general characteristics are mandatory, such as a minimum/maximum number of gates, approximate distances, and interval durations of 90–110 s (ICF, 2021), great variability still occurs from race to race. Therefore, we did not include studies with specific performance trials. Some of the eligible studies tried to follow such recommendations in both whitewater (Messias et al., 2015; Vieira et al., 2015; Ferrari et al., 2017) and flatwater (Macdermid et al., 2019) tasks; nonetheless, considerable variability was also seen in these. The idea of creating a standard performance protocol—identical courses, obstacles, eddies, waves, and stoppers—for this sport with strong ecological validity is likely utopic because canoe slalom competition preserves the unpredictability (Nibali et al., 2011). Thus, others opted for a different strategy and focused on a particular technical skill (Baláš et al., 2020) or used the results from slalom championships (Hunter et al., 2008; Bielik et al., 2019, 2020). Overall, it is too early to affirm or suggest the most suitable strategy to evaluate canoe slalom performance, but without further investigations, this issue will not be resolved.

### Limitations and Strengths

The results of this systematic review should be understood in light of these limitations. Few studies were eligible, and the outcomes presented must be confirmed in further reports. Moreover, the mechanical, physiological, and technical assessments were conducted mostly with male participants. A recent study by Tilden et al. (2021) identified differences in stroke techniques between male and female athletes in international competitions. Nevertheless, future studies assessing the mechanical, physiological, and technical components of female slalom athletes along with their performance are still necessary. We did not perform a meta-analysis on the studies due to the large variability of the protocols adopted for assessing mechanical, physiological, and technical parameters, as well as the distinct outcomes; such variability would have resulted in heterogeneity of the results. Additionally, some eligible studies adopted simulated tasks in both whitewater and flatwater courses for measuring performance, while others considered data from international championships. There is a large strategy variation used to negotiate the gates among the top canoe slalom athletes. This is likely associated with variations in training, equipment, technical ability, strength, decision-making skills, and course perceptions (Hunter et al., 2008). The strengths of this study include the high-quality assessment of the eligible reports and the high level of athletes included in most of these studies. Finally, this is the first systematic review performed in canoe slalom studies.

## Conclusion

This study concludes that mechanical, physiological, and technical factors may play important roles in canoe slalom performance. Further studies are recommended on these issues along with slalom performance assessments, which deserve significant attention in terms of standardization and ecological validity. Together, these studies should confirm the presented outcomes and advance the science surrounding this sport, helping coaches and athletes throughout the training period and, most importantly, in competitions.

## Data Availability Statement

The raw data supporting the conclusions of this article will be made available by the authors, without undue reservation.

## Author Contributions

LM: proposal of ideas, the conception, design of the work, acquisition, interpretation and analysis of data, the writing of the main manuscript text, and preparing figures and tables. IR and AG: acquisition, interpretation and analysis of data, the writing of the main manuscript text, and preparing figures and tables. VB: writing of the main manuscript text and preparing figures and tables. CG and FM-G: proposal of ideas, the conception, interpretation and analysis of data, and the writing of the main manuscript text. All authors contributed to the article and approved the submitted version.

## Funding

The authors thank the Fundação de Amparo à Pesquisa do Estado de São Paulo (FAPESP—Proc. 2012/06355-2, Proc. 2010/17134-1 and Proc. 2021/12447-6) and VEGA (1/0260/21) for financial support.

## Conflict of Interest

The authors declare that the research was conducted in the absence of any commercial or financial relationships that could be construed as a potential conflict of interest.

## Publisher's Note

All claims expressed in this article are solely those of the authors and do not necessarily represent those of their affiliated organizations, or those of the publisher, the editors and the reviewers. Any product that may be evaluated in this article, or claim that may be made by its manufacturer, is not guaranteed or endorsed by the publisher.
